# Micro-structured polymer fixed targets for serial crystallography at synchrotrons and XFELs

**DOI:** 10.1107/S2052252523007595

**Published:** 2023-09-20

**Authors:** Melissa Carrillo, Thomas J. Mason, Agnieszka Karpik, Isabelle Martiel, Michal W. Kepa, Katherine E. McAuley, John H. Beale, Celestino Padeste

**Affiliations:** a Paul Scherrer Institut, Forschungsstrasse 111, 5232 Villigen, Switzerland; b Department of Chemistry, University of Basel, Mattenstrasse 24a, 4002 Basel, Switzerland; c Swiss Nanoscience Institute, Klingelbergstrasse 82, 4056 Basel, Switzerland; d Institute of Polymer Nanotechnology (INKA), FHNW University of Applied Sciences and Arts Northwestern Switzerland, School of Engineering, Klosterzelgstrasse 2, 5210 Windisch, Switzerland; Uppsala University, Sweden

**Keywords:** fixed targets, serial crystallography, free-electron lasers, micro-structured polymer chips, apertures, time-resolved studies, sample delivery

## Abstract

Fixed targets are a popular form of sample-delivery system used in serial crystallography at synchrotron and X-ray free-electron laser sources. They offer a wide range of sample-preparation options and are generally easy to use. The supports are typically made from silicon, quartz or polymer. Of these, currently, only silicon offers the ability to perform an aperture-aligned data collection where crystals are loaded into cavities in precise locations and sequentially rastered through, in step with the X-ray pulses. The polymer-based fixed targets have lacked the precision fabrication to enable this data-collection strategy and have been limited to directed-raster scans with crystals randomly distributed across the polymer surface. Here, the fabrication and first results from a new polymer-based fixed target, the micro-structured polymer fixed targets (MISP chips), are presented. MISP chips, like those made from silicon, have a precise array of cavities and fiducial markers. They consist of a structured polymer membrane and a stabilization frame. Crystals can be loaded into the cavities and the excess crystallization solution removed through apertures at their base. The fiducial markers allow for a rapid calculation of the aperture locations. The chips have a low X-ray background and, since they are optically transparent, also allow for an *a priori* analysis of crystal locations. This location mapping could, ultimately, optimize hit rates towards 100%. A black version of the MISP chip was produced to reduce light contamination for optical-pump/X-ray probe experiments. A study of the loading properties of the chips reveals that these types of fixed targets are best optimized for crystals of the order of 25 µm, but quality data can be collected from crystals as small as 5 µm. With the development of these chips, it has been proved that polymer-based fixed targets can be made with the precision required for aperture-alignment-based data-collection strategies. Further work can now be directed towards more cost-effective mass fabrication to make their use more sustainable for serial crystallography facilities and users.

## Abstract 2

The micro-structured polymer chip is a new polymer fixed-target support for serial crystallography experiments at synchrotrons and X-ray free-electron lasers; these micro-structured supports contain a precise array of apertures that allow rapid aperture-alignment-based data-collection strategies. Here, the chip-fabrication process is presented and discussed, as well as an analysis of ideal crystal-loading parameters and data quality.

## 1. Introduction

Serial crystallography at X-ray free-electron lasers (XFELs) and synchrotron light sources, called serial femtosecond crystallography (SFX) and serial synchrotron crystallography (SSX), respectively, has proved to be a successful and robust methodology. The method has perhaps best been exemplified by the regular practice of time-resolved crystallography (Redecke *et al.*, 2013 ▸; Barends *et al.*, 2015 ▸; Ishigami *et al.*, 2019 ▸; Nogly *et al.*, 2018 ▸; Weinert *et al.*, 2019 ▸), but also in damage-free or low-dose structures (Fukuda *et al.*, 2016 ▸; Halsted *et al.*, 2018 ▸; Barnes *et al.*, 2019 ▸; Moreno-Chicano *et al.*, 2019 ▸) and micro *in cellulo* crystallography (Sawaya *et al.*, 2014 ▸; Jakobi *et al.*, 2016 ▸; Boudes *et al.*, 2017 ▸). Excitingly, serial crystallography continues to evolve and find new triggering technologies (Olmos *et al.*, 2018 ▸; Mehrabi *et al.*, 2019 ▸) and hybrid methods (Kern *et al.*, 2018 ▸; Rabe *et al.*, 2021 ▸; Kepa *et al.*, 2022 ▸).

**Figure 1:**
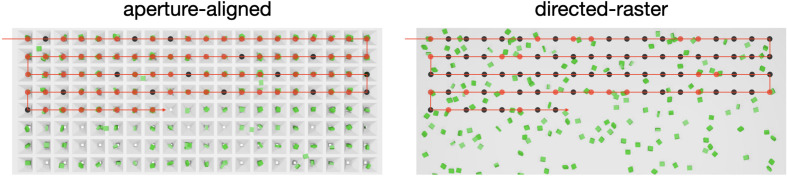
A schematic of the different data-collection types supported by fixed targets. The two methods of data collection performed are aperture-aligned and directed-raster fixed targets. Aperture-aligned fixed targets allow for crystals to be deposited in precise locations, whereas directed-raster fixed targets have the crystals randomly distributed throughout the surface of the chip.

To cater for these different experiments, a wide variety of delivery methods have been developed (Martiel *et al.*, 2019 ▸; Chen *et al.*, 2019 ▸). Amongst these, fixed targets, or chips, have proved to be a robust and reliable approach (Barends *et al.*, 2022 ▸). Fixed targets can enable two types of data collection (Fig. 1 ▸): aperture aligned, where crystals are loaded into precisely positioned cavities at known locations, and sequentially exposed or directed raster, where a raster grid is defined over crystals in completely random locations. The utilization of the aperture-aligned mode has only been possible with the utilization of precision silicon fabrication techniques to create micro-structured solid supports (Oghbaey *et al.*, 2016 ▸; Mehrabi *et al.*, 2020 ▸) and stage motion (Sherrell *et al.*, 2015 ▸).

Although there is still considerable variation around different facilities, silicon fixed-target supports (∼30 × 30 mm with ∼25 000 etched cavities) are now commonly used in at least three facilities: Time-Resolved Experiments with Crystallography (T-REXX) (https://www.embl.org/groups/macromolecular-crystallography/p14-eh2/), PETRA III, Ger­many; ID29 (https://www.esrf.fr/id29), ESRF–EBS, France; and I24 (https://www.diamond.ac.uk/Instruments/Mx/I24.html), Diamond Light Source (DLS), UK. Due to the size of the chips, precise cavity placement must also be coupled to precise motion and alignment strategies. Silicon wafers offer a viable means to these ends. Silicon is rigid, inert and can be precisely chemically etched. Any diffraction spots given by the silicon crystals are more aesthetically disagreeable rather than deleterious to the detector or experiment. The silicon chip has, therefore, been essential to the establishment of aperture-aligned methods in the serial crystallography toolbox.

However, the use of silicon also has a number of disadvantages. Though rigid, even when only 50 µm thick, it is also highly brittle and prone to fracture. Silicon is also opaque, making it difficult to know *a priori* how well crystals have been loaded into the cavities. Currently, silicon chips are also posing a significant cost to users. Given these issues, it is worthwhile pursuing alternative materials as the basis for such micro-structured supports. Here, polymers offer an advantageous substitute, provided a comparable means of cavity fabrication and precision can be achieved.

The use of polymer supports is not new in protein crystallography. Loops and meshes, made from nylon or Kapton, are common mounts in cryo-crystallography. Samples either encased or surrounded by pieces of thin film are also routine under cryo-conditions or at room temperature (Huang *et al.*, 2015 ▸; Axford *et al.*, 2016 ▸; Baxter *et al.*, 2016 ▸) and enable the directed-raster mode. Recently, more complex micro-structured supports, specifically for room-temperature samples, have started to become available. The Crystallography Sample Supports (MiTeGen) are mounted on standard SPINE pins but currently lack the larger area format of the silicon chips (Illava *et al.*, 2021 ▸). Large-area polymer supports have been developed but are also currently limited to directed-raster data-collection strategies. These range from simple thin-film sandwiches (Doak *et al.*, 2018 ▸; Rabe *et al.*, 2020 ▸; Park *et al.*, 2020 ▸; Lee *et al.*, 2020 ▸) [also possible in using quartz (Ren *et al.*, 2018 ▸)] to more regular structures encased within film (Lee *et al.*, 2019 ▸; Martiel *et al.*, 2021 ▸; Nam *et al.*, 2021 ▸; Sherrell *et al.*, 2022 ▸) or resting on film (Kepa *et al.*, 2022 ▸). A method to precisely fabricate a regular array of cavities in a polymer film is still lacking, resulting in the lack of polymer chips in aperture-aligned data-collection modes.

Here, micro-structured polymer (MISP) chips are presented. The supports retain very high precision in cavity fabrication, similar to silicon-based chips; however, their raw materials are a fraction of the cost of silicon, and offer greater flexibility in design and cavity shape. Their stabilizing frame proves to be beneficial in increasing the hit rate and sample efficiency. They are also more robust, making them easier to handle and user friendly when compared with the silicon chips. The chips were tested at the SwissFEL Cristallina experimental station using the serial with solid-support macromolecular crystallography (MX) (SwissMX) endstation. Due to the success of the chips in the commissioning experiments, they will be the principal workhorse fixed target of the endstation.

## 2. Methods

### 2.1. Preparation of silicon masters

The fabrication of MISP chips begins with the preparation of a silicon master from a double-sided polished 4” silicon wafer coated with 100 nm silicon nitride (Si_3_N_4_). The wafer is spin coated with a photosensitive resist (Microposit S1813 G2, Micro Resist Technology) at 4000 r min^−1^ and soft baked for 90 s at 115°C. The design for the MISP chips is created on *KLayout* software (https://www.klayout.de/) and transferred onto the wafer using laser lithography (Heidelberg Instruments – DWL 66^+^). The wafer is then placed in a developer bath for 1 min to dissolve away the regions that were exposed to light. The structure is transferred onto the Si_3_N_4_ through reactive ion etching (RIE) (Oxford Instruments – PlasmaPro 100: 5 standard cm^3^ min^−1^ O_2_, 40 standard cm^3^ min^−1^ CHF_3_, 2 min). Subsequently, the wafer is etched through with potassium hydroxide (KOH etching at 80°C, 1.5 h). The wafer is then rinsed with water, and the remaining silicon nitride is removed using RIE. In preparation for the following steps, the silicon wafer’s surface is activated in oxygen plasma (Oxford Instruments – PlasmaPro 80: 20 standard cm^3^ min^−1^ O_2_, 150 W, 2 min) and then coated with a fluorosilane anti-sticking layer (Schift *et al.*, 2005 ▸).

### 2.2. Fabrication of working stamps

A glass wafer is prepared by surface activation with oxygen plasma (Oxford Instruments – PlasmaPro 80: 20 standard cm^3^ min^−1^ O_2_, 150 W, 2 min), spin coating with Ormoprime 08 (Micro Resist Technology, 4000 r min^−1^, 60 s) and then hard baking for 5 min at 150°C. Ormostamp (Micro Resist Technology) is dispensed on the silicon master and covered by the glass wafer, waiting until all cavities are filled. The assembly is then exposed to ultraviolet C (UV-C) radiation (compact UV–LED chamber BSL-01 – Opsytec Dr Gröbel: 210 mW cm^2^, 2 min) and hard baked on a hotplate for 30 min at 130°C. The samples are then unmoulded, resulting in a glass/Ormostamp working stamp used for membrane production. The surface is activated through oxygen plasma (Oxford Instruments – PlasmaPro 80: 20 standard cm^3^ min^−1^ O_2_, 20 W, 20 s) and finally coated with a fluorosilane anti-sticking layer.

### 2.3. Transparent COP and black COC

Imprints were performed on transparent 50 µm cyclic olefin polymer (COP) (microfluidic ChipShop) and black cyclic olefin copolymer (COC) film. Black COC films were manufactured in-house by first dispersing 1.7 g of carbon black (Acetylene Carbon Black Li400, UBIQ, Japan) in 100 ml toluene under ultrasound agitation for 1 h. Then, 17.0 g of COC granules (TOPAS 8007X10, TOPAS Advanced Polymers, Oberhausen, Germany) were added and dissolved within 24 h under constant stirring and heating to 60°C. The resulting viscous solution was spread out on glass plates using a ZAA automatic film applicator (Zehntner, Switzerland; barrel applicator with 500 µm gap, 15 mm s^−1^) and dried at 60°C, yielding films with thicknesses in the range of 52–55 µm.

### 2.4. Fabrication of fixed targets

Fixed-target membranes were produced by hot embossing the working stamp into a COP or COC film, backed by 50–250 µm of Kapton and 0.1–1.0 mm of Teflon, using a Jenoptic HEX 03 hot press (180°C, 300 N, 15 min). After cooling down, the structured films were released from the stamp and underwent plasma activation (Oxford Instruments – PlasmaPro 80: 20 standard cm^3^ min^−1^ O_2_, 150 W, 2 min) shortly before attachment of the frames. Acrylic frames were designed using the software *Fusion 360* (AutoDesk, https://www.autodesk.com/products/fusion-360) and 3D printed on a ProJet MJP 2500 Series 3D Printer. Frames were then glued onto the membrane with epoxy glue and left overnight to harden. Afterwards, they were cut and removed from the excess COC and COP film.

### 2.5. Sample preparation

Hen egg-white lysozyme (HEWL) (Sigma–Aldrich) was dissolved to a final concentration of 50 mg ml^−1^ in 0.1 *M* sodium acetate, pH 3.0. The protein concentration was measured using a NanoDrop One UV–Vis spectrophotometer (Thermo Scientific) at 280 nm using an extinction coefficient of 37470 *M* ^−1^ cm^−1^. Different-sized HEWL micro-crystals were obtained by varying the protein and crystallization-buffer concentrations (Table 1 ▸). The crystallization buffer was 0.1 *M* sodium acetate, pH 3.0, 28%(*w*/*v*) sodium chloride, 8%(*w*/*v*) PEG 6000. To make the micro-crystal slurry, 500 µl of protein and crystallization buffer was mixed in a 1.5 ml centrifuge tube, and immediately vortexed for 10 s. The centrifuge tube was then left stationary overnight (∼16 h) at 20°C. The number of crystals and their sizes were then estimated using a hemocytometer (Hausser Scientific) and a HIROX RH-2000 digital microscope with a MXB-5000REZ zoom lens.

### 2.6. Sample loading

A loading station was built for sample loading into the MISP chips. It consists of a loading platform, a vacuum pump to extract the excess mother liquor, and a humidity stream to keep the crystals from dehydrating during preparation. Vacuum-pump suction was controlled with a valve, which provided control over the intensity and timing of excess-mother-liquor extraction (see Fig. 9).

The loading platform was designed on *Fusion 360* and 3D printed on a ProJet MJP 2500 Series 3D printer [Fig. 9(*b*)]. This loading platform provides a support for the membrane of the MISP chips whilst under suction, prolonging the lifespan of the MISP chips. The loading surface contains a gasket cut from a polydimethylsiloxane (PDMS) sheet, serving as a vacuum seal.

The samples were loaded by pipetting the crystal slurry onto the top surface of the chip when placed on the holder, and removing the excess mother liquor by applying a vacuum. Loading was performed under a constant humidity stream. The chip was then transferred to a chip holder (designed on *Fusion 360* and 3D printed on a ProJet MJP 2500 Series 3D printer) and enclosed with two layers of 6 µm Mylar film [Fig. 9(*c*)]. Typically, five MISP chips were loaded, one after another, placed in a chamber, and kept at a constant 80% humidity before the entire chamber was transported to a hutch. Previous work indicated that samples could be stored in a humidified environment for up to seven days prior to measurement without apparent loss of diffraction quality (Huang *et al.*, 2022 ▸).

### 2.7. Data collection

Data were collected over three beam times in May, September and October 2022 at the Cristallina experimental station of SwissFEL using the SwissMX endstation. Consistent self-amplified spontaneous-emission (SASE) parameters were achieved across all three beam times with pulse width, peak energy and repetition rate set to 35 fs [20 fs root mean square (RMS)], ∼40–50 µJ and 100 Hz, respectively. The achieved photon energy varied slightly between beam times, with central energies of 11.36 ± 0.05, 11.30 ± 0.05 and 11.26 ± 0.05 keV, respectively.

All data were collected in air but with scatter guards upstream and downstream of the sample position, such that scattering from only a 15 mm section of the beam path could reach the detector. The detector distance varied over the three beam times from 150 to 110 mm, which corresponded to an achievable resolution difference of 1.65–1.35 Å on an 8 Mpixel JUNGFRAU detector.

The MISP chips were designed with fiducials in known locations to assist aperture alignment. The *x*/*y*/*z* coordinates of these fiducials were recorded with respect to the X-ray beam. The locations of cavity apertures could then be inferred by a coordinate transformation (Sherrell *et al.*, 2015 ▸). Due to the slight shrinkage of chips from their design specifications during the hot-embossing process, the intra-aperture distances needed to be scaled. To do this on a per-chip basis, the mean fiducial distance (FiD_obs_) was determined by averaging the distance between the four recorded motor positions of each fiducial. The ratio between the measured fiducial distance (FiD_obs_) and the designed fiducial distance (FiD_calc_) could then be used as a ‘shrinkage factor’ to be applied to all the other intra-aperture distances: 

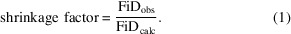




### 2.8. Data processing and analysis

*CrystFEL* (White *et al.*, 2012 ▸) version 0.10.0 was used to analyse the data. The algorithms *peakfinder8* and *XGANDALF* (Gevorkov *et al.*, 2019 ▸) were used to find spots and index images, respectively, with the following settings: –threshold = 10, –int-radius = 3,4,7, –min-snr = 5.0, –min-peaks = 10 and –min-pix-count = 2. Data from the resulting stream files were then individually extracted for downstream analysis. To calculate CC_1/2_ values, ∼30 000 integrated patterns for each HEWL crystal size were merged using *partialator* with –model = unity and –iterations = 1.

Initial phases were calculated using *Phaser* (McCoy *et al.*, 2007 ▸) and the previously solved HEWL structure, PDB ID 6abz (Seraj & Seyedarabi, 2020 ▸), as a search model. Refinement was completed using *Phenix* (Liebschner *et al.*, 2019 ▸) and model building was performed using *Coot* (Emsley & Cowtan, 2004 ▸). Final refinement statistics for all structures are shown in Table 3. Figures were made using *PyMOL* (Schrödinger, LLC, 2015 ▸).

## 3. Results

### 3.1. Chip fabrication

#### 3.1.1. Membrane manufacturing

**Figure 2:**
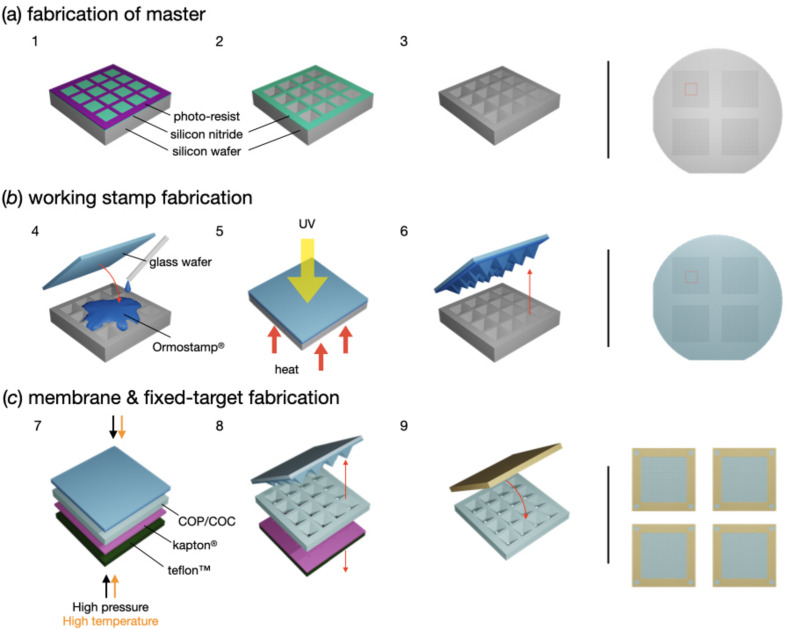
A fabrication overview of a MISP chip. (*a*) (1) The design of the MISP chip is translated into a silicon nitride layer on a silicon wafer using laser lithography and RIE. (2) KOH etching forms the inverted pyramidal wells at the surface of the silicon wafer. (3) The remaining silicon nitride is removed using RIE, resulting in the master stamp. (*b*) (4) Ormostamp is pipetted onto the master and a prepared glass wafer is slowly placed on the top. (5) By applying UV light and heat, the Ormostamp hardens and moulds into the silicon-wafer wells. (6) The working stamp is lifted off resulting in pyramids on the working stamp. (*c*) (7) The working stamp is used to imprint the cavities into the COC or COP film through hot embossing. (8) The film is demoulded from the working stamp. (9) Frames are glued onto the film resulting in the final product of the polymer fixed targets. Images on the right show the final product of the master stamp, working stamp and fixed targets.

The procedure to manufacture the structured polymer membrane, representing the central part of the MISP chips, is shown in Fig. 2 ▸. It starts with the fabrication of a silicon master, taking advantage of well established and highly precise silicon micro-fabrication techniques. More specifically, cavities shaped as inverted pyramids are wet etched into a silicon 〈100〉 wafer [Fig. 2 ▸(*a*)]. A silicon nitride layer structured with laser lithography and RIE serves as a hard mask during the anisotropic KOH etching. This follows, due to preferential etching in the parallel direction, exactly with the silicon 〈100〉 planes, resulting in an angle of the sidewalls of 54.74° with respect to the wafer surface. This angle defines the exact geometry of the pyramids (relation of the base plane and height) and results in constraints for the membrane design (see below).

From the master structure, a working-stamp copy is made using OrmoStamp, a UV-curable hybrid material that enables high-fidelity replication of micro-structures and is suitable as a material for nano-imprint lithography [Fig. 2 ▸(*b*)]. This working stamp is then hot embossed into a film of a thermoplastic material such as COC or COP [Fig. 2 ▸(*c*)]. The exact balance of structure sizes and film thickness as well as material combinations used as backing layers play important roles in obtaining cavities in the films with defined apertures at the bottom. As backing for the COC or COP film, a polyimide film is placed directly underneath, which does not soften at the process temperature. This is followed by Teflon, which is relatively soft and can level out forces on a larger scale. Pyramid heights must exceed the film thickness by more than the intended punching-through height, as the polymer displaced in the process results in an apparent increase in film thickness (see Appendix *A*1[App appa]). For instance, when using 50 µm thick COP films and pyramid structures of 100 µm side length (70.80 µm height) at 120 µm pitch, the formed holes had dimensions of ∼5–7 µm, indicating that only the top 4–5 µm of the pyramids were punching through the film.

#### 3.1.2. Parameterization of chips

**Figure 3:**
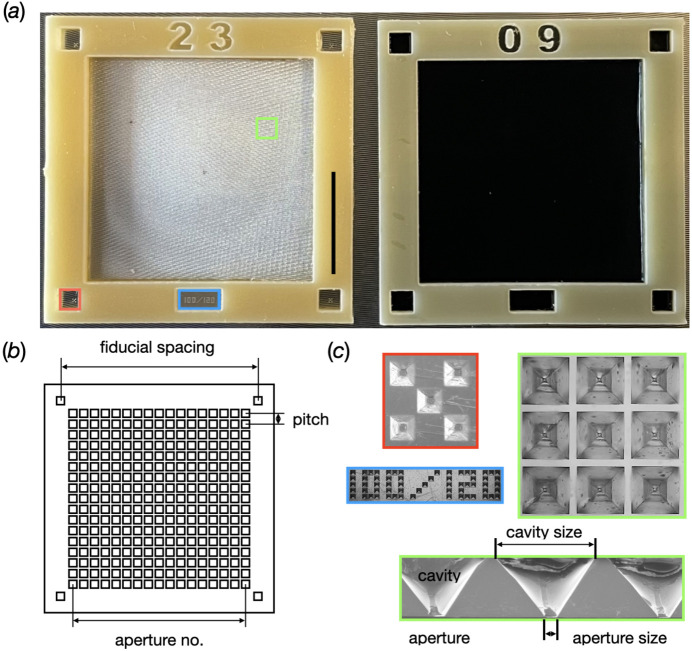
An overview of the MISP chips. (*a*) The two current versions of the MISP chip with transparent COP and black COC membrane. The black scale bar shown on the frame of the transparent COP MISP chip represents 10 mm. (*b*) The parameters used to define the chips. (*c*) Magnified fiducials (red box), cavities (green box), cavity profiles (green) and label.

MISP chips are globally defined by three parameters [Fig. 3 ▸(*b*)]: (i) the fiducial spacing, the distance between corner fiducials; (ii) the aperture pitch, the distance between adjacent aperture centres; and (iii) the number of apertures, the number of openings per row of apertures.

Initial designs featured 12.5 mm fiducial spacing, 120 µm aperture pitch and an array of 78 × 78 apertures. This was done to prototype the manufacturing process as well as the fiducial-alignment software. After the first commissioning of the SwissMX instrumentation and confirmation that the MISP chips were compatible with a fiducial-alignment based data collection, the MISP chips were enlarged to increase the total number of available apertures per chip. These larger chips have 23.0 mm fiducial spacing, 120 µm aperture pitch and 172 × 172 apertures, giving an increase in total aperture number from 6084 to 29 584 per chip.

The MISP chips were developed on transparent COP and black COC film with thicknesses of 50 ± 1 and 52 ± 3 µm, respectively (see Appendix *A*1[App appa]). There was good agreement between the original layout and the structured replicates in both the transparent and black films. However, a linear shrinkage of the dimensions of ∼1% was determined on both types of films, which was assigned to a contraction of the material when cooling down from the embossing temperature. The aperture sizes in the centre of the membrane area varied between the two film types [Fig. 3 ▸(*b*)]. For the transparent chips, they were consistently between 5 and 7 µm. On the black chips, they were more dependent on the batch of film and fluctuated in a range from 4 to 12 µm.

For both the transparent and black chips, there were consistent variations in aperture sizes around the edges of the membrane. This was due to the build-up of excess material shifting during hot embossing. The rows on the edge of the cavity arrays consistently showed larger apertures. For the transparent and black chips, the maximum observed apertures measured ∼13 µm and ∼20 µm, respectively (for details see Appendix *A*1[App appa]). However, issues that may have arisen from this phenomenon were limited, since most of the enlarged apertures were covered when the chip frames were glued onto the film surface.

#### 3.1.3. Overcoming chip bending

In the first round of chip making, the chips showed a pronounced bending orthogonal to the membrane, sometimes as large as 100–200 µm from base to apex. This was found to be caused by the fixation of the frame to the COP and COC membrane. The fixation was performed in the same manner as reported earlier (Karpik *et al.*, 2020 ▸), *i.e.* by directly printing a polylactic acid (PLA) frame onto the COP film using a filament printer. However, direct printing caused the membrane to heat, and the bending was a result of the different thermal-expansion coefficients of the film and PLA filament. To overcome this issue, frames were 3D printed on a multi-jet printer using acrylic and then fixed onto the membrane with epoxy glue. This change rectified the pronounced bending.

### 3.2. Loading analyses

Loading of crystals onto the MISP chips was carried out using a humidity sample-loading station constructed specifically for the MISP chips [see Appendix *A*2[App appa], Fig. 9(*a*)]. This setup provided a humidity stream over the sample-loading platform [Appendix *A*2[App appa], Fig. 9(*b*)], which was attached to a vacuum pump. This allowed for the excess mother liquor to be removed while keeping the crystals hydrated. A chip was placed onto the sample-loading platform and then the crystal slurry was pipetted onto the surface of the chip. The valve to the pump was then opened and excess mother liquor was removed. Once complete, the chip was placed into a chip holder, sealed [see Appendix *A*2[App appa], Fig. 9(*c*)], and placed in a humidity chamber.

**Figure 4:**
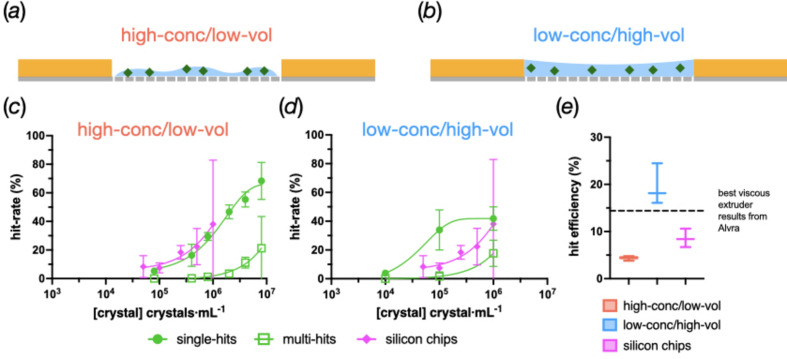
A comparison of different loading methods. (*a*) and (*b*) Schemes showing the practical difference between the loading methods. (*a*) In high-conc/low-vol experiments, low volumes of highly concentrated slurries were tested. (*b*) In low-conc/high-vol experiments, the reverse was true; higher volumes of less concentrated slurries were used. (*c*) and (*d*) Graphs plotting aperture hit rates as a function of crystal concentration for 25 µm HEWL crystals for the high-conc and low-conc methods, respectively. The aperture hits have been divided into single- and multi-hits to indicate whether one or more than one crystal was found in the well. Data obtained from 25 µm insulin crystals deposited on silicon chips at a synchrotron (Davy *et al.*, 2019 ▸) have also been plotted for comparison. (*e*) An assessment of the loading efficiency given as a percentage of the absolute number of crystals used versus exposed. The single-hit data from silicon chips (Davy *et al.*, 2019 ▸) have also been plotted, and an estimate of the efficiency of a viscous extruder, with comparably sized crystals, from a beam time at the SwissFEL Alvra endstation.

Two different strategies were used to load the sample onto the chips. Initially, crystals were loaded with just enough solution such that the slurry could be spread over the surface of the chip [Fig. 4 ▸(*a*)]. This high-concentration/low-volume (‘high-conc/low-vol’) strategy proved effective for 25 µm HEWL crystals and was comparable, in terms of hit rate, to silicon chips with 25 µm insulin crystals loaded in a similar manner [Fig. 4 ▸(*c*)] (Davy *et al.*, 2019 ▸).

However, moving to a low-conc/high-vol method was found to be beneficial for the sample deposition. Here, a larger volume of solution was used to load the same number of crystals such that the entire well surrounded by the acrylic frame was filled with liquid [Fig. 4 ▸(*b*)]. This change meant that a much lower crystal concentration was required to achieve comparable hit rates [Fig. 4 ▸(*d*)]. It also proved to drastically improve the sample efficiency.

Fig. 4 ▸(*e*) shows the absolute hit rate with respect to the actual number of crystals that were loaded onto the chips. The low-conc/high-vol strategy shows an approximate fourfold and twofold increase in sample efficiency compared with the high-conc/low-vol method and data obtained from silicon chips, respectively. An estimated sample efficiency is also shown from a viscous extruder experiment measured at Alvra (SwissFEL, Paul Scherrer Institut). The low-conc/high-vol method is on a par with this as well. All subsequent data shown in this article were collected using this low-conc/high-vol loading method.

### 3.3. The effect of crystal size and concentration on hit rate

**Figure 5:**
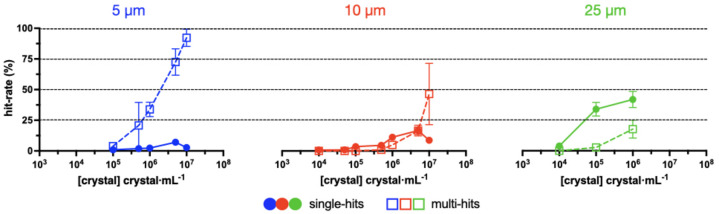
The effect of HEWL crystal size and concentration on hit rate. The mean and 95% confidence interval for each data point have been plotted. Each data point was based upon at least three measurements. Single- and multi-hits denote either single or multiple crystal lattices observed in each well, respectively.

It became clear from the first experiments that crystal size was a significant factor in determining the eventual hit rate. To test this systematically, 5, 10 and 25 µm HEWL crystals were prepared. Chips were then loaded with different concentrations of these crystals and the hit rates were recorded (Fig. 5 ▸). Apertures that gave rise to hits have been split into either single hits or multi-hits, signifying that either only a single diffraction pattern or overlaid patterns were observed, respectively.

The results indicate that it is very challenging to obtain a high rate of single hits (>30%) for crystals of ≤10 µm. It appears that, for smaller crystals, a higher concentration is required to locate crystals in the chip apertures. This can best be observed by comparing the hit rates at a concentration of 1 × 10^5^ crystals ml^−1^. This is enough to obtain a hit rate of over 30% in the 25 µm crystals, but only 2% in the 10 µm crystals and 1% in the 5 µm crystals. As the concentration in the 5 and 10 µm crystals is increased, the hit rates do improve, but both in the number of single- and multi-hits observed. This effect is particularly pronounced for the 5 µm crystals.

### 3.4. Comparing calculated and observed hit rates

One of the benefits of fixed targets, when compared with other delivery methods, is that it should be possible to determine crystal distribution before data collection. The logical extension of this idea is ‘chip mapping’ (Oghbaey *et al.*, 2016 ▸), where all crystal locations on the chip are known, such that empty apertures can be avoided and close to 100% hit rates obtained. However, crystals loaded on opaque silicon chips have proved difficult to see under a microscope and, so far, could only be spectroscopically observed when the protein crystals were coloured (Oghbaey *et al.*, 2016 ▸).

**Figure 6:**
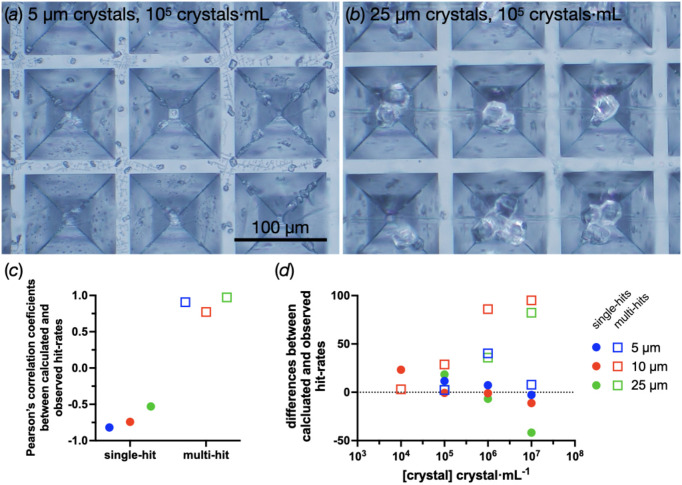
The opportunities and problems with observing crystal locations under a microscope. (*a*) and (*b*) Wells from chips loaded with 5 and 25 µm crystals at 1 × 10^5^ crystals ml^−1^, respectively. (*c*) The correlation between calculated (counted under a microscope) and observed (diffraction detected) hit rates for the 5, 10 and 25 µm crystals. (*d*) The residual differences between the observed and calculated hit rates.

The clear COP chips have changed this. Crystals loaded are now clearly observable under a standard microscope [Figs. 6 ▸(*a*) and 6 ▸(*b*)]. These images give us a better understanding of the low hit rates when using 5 and 10 µm crystals. Smaller crystals appear to be scattered throughout the surface of the chip rather than being drawn into the apertures at the centre of the cavities.

Given the fact that crystals are clearly visible on the clear chips, it should be possible to check the loading of the fixed target once it has been prepared, giving users real-time feedback on sample preparation. However, the natural excitement towards this opportunity should be tempered as the human eye and mind can be easily fooled. Fig. 6 ▸(*c*) shows Pearson’s correlation coefficient between the ‘microscope calculated’ and diffraction-observed hit rates for both single- and multi-hits, but even a favourable interpretation of these data with regard to the multi-hit correlation should be tempered. Fig. 6 ▸(*d*) shows the differences between the calculated and observed rates in terms of real numbers, showing that even positive correlations can be widely inaccurate.

### 3.5. Diffraction quality

**Figure 7:**
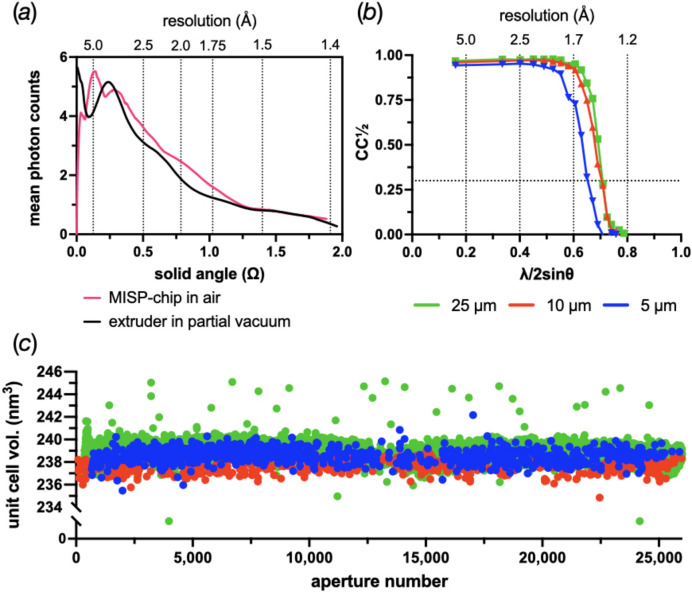
Data-quality indicators from chip data collections. (*a*) A comparison of the total background observed between the MISP chips collected at Cristallina and the viscous extruder collected in the Alvra gas chamber with 200 mbar He. The background is shown as a radial integral given in mean photon counts. The solid angle shown has been limited to the effective size of a 4 Mpixel detector. The total background from the MISP chip will also include contributions from the air, sealing films and sample. (*b*) CC_1/2_ plots for the 25, 10 and 5 µm HEWL crystals. Each is based on ∼30 000 integrated and merged images. The 0.3 criterion line has been shown. (*c*) The unit-cell volume plotted as a function of aperture number for representative chips loaded with 25, 10 and 5 µm HEWL crystals.

**Figure 9:**
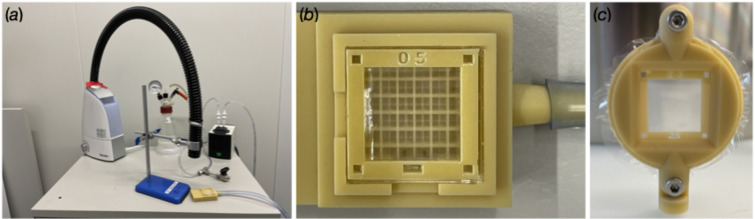
Essential items for the MISP chip. (*a*) The humidity sample-loading station is made to prevent crystals from dehydrating during preparation. (*b*) The sample-loading platform is designed to extract excess mother liquor from the MISP chip while providing support on the membrane. (*c*) The sample holder is designed to seal the loaded crystals on the MISP chip from dehydration and is designed for mounting onto the goniometer at SwissMX.

Finally, it is worth looking at the quality of the data that can be obtained when using the MISP chips. Unlike other delivery methods, fixed targets will usually place solid material in the path of X-rays; therefore, the background is a serious consideration. Fig. 7 ▸(*a*) shows the mean radial integral of the detector from a MISP chip in air collected at 11.34 keV with a pulse energy of ∼50 µJ. For comparison, a radial integral from a 50 µm viscous jet collected in SwissFEL’s Alvra gas chamber at 200 mbar He and 12.4 keV is also shown. Pleasingly, both are very comparable. In the near future, SwissMX will also be capable of data collections in a helium environment, reducing background noise even further.

Fig. 7 ▸(*b*) shows CC_1/2_ plots for the three HEWL samples used to evaluate the chip loading. There is a gradual decline in the quality of the data, based on CC_1/2_, as a function of the crystal size. The difference is almost negligible between the 25 and 10 µm crystals but is quite pronounced for the 5 µm crystals. A drop in resolution using the 0.3 criterion from 1.41 to 1.54 Å was determined.

To see if there was any degradation of crystal quality as the chip was exposed to the beam, the unit-cell volume was calculated for every single crystal hit. The volumes were then plotted as a function of the aperture number to see if any change was observable. No change was detected throughout the five minutes of collection time for either the 25, 10 or 5 µm HEWL crystals; typical results are shown in Fig. 7 ▸(*c*).

When working with fixed targets, one must keep in mind that the crystals are being placed onto a surface, which can raise concern to preferential orientation. Evaluation of the crystal orientation (Appendix *A*4[App appa]) did indeed show a certain degree of preferential orientation, but the small magnitude did not affect the structure determination in the present case. However, preferential orientation is expected to be more pronounced for highly anisotropic crystal shapes such as platelets and needles, and needs to be evaluated for individual cases.

Structures were solved for all three different crystal sizes (Appendix *A*5[App appa]), with a resolution of 1.54 Å for the 5 µm crystals, and 1.40 Å for the 10 and 25 µm crystals, and they are in good agreement with published room-temperature HEWL data.

## 4. Discussion

The MISP chips are an attempt to combine the precision of the silicon micro-structured chips (Oghbaey *et al.*, 2016 ▸; Ebrahim *et al.*, 2019 ▸; Mehrabi *et al.*, 2020 ▸) with the ease of use and low cost of the polymer supports. By creating a precise array of apertures in a polymer, aperture-alignment-style data-collection strategies can be performed. Like many such endeavours aimed at trying to capture all that is best and none that is worst, some compromises were required.

### 4.1. MISP-chip characterization

The first priority was to devise a fabrication process that had the precision to facilitate aperture-alignment-style data collections. This was achieved using a combination of silicon micro-fabrication and hot-embossing techniques. The first prototype was 8 × 8 mm (4356 cavities), then scaled to 12.5 × 12.5 mm (7056 cavities) and currently 23 × 23 mm (29 584 cavities) from fiducial to fiducial. The increase in size had no side effects on the precision.

The hot-embossing step during film-membrane fabrication (Fig. 2 ▸) slightly affects the precision of the MISP chip, in that the film contracts by ∼1% when cooling down from the imprinting temperature. Once shrinkage is determined, all intra-aperture distances can be appropriately scaled. This information is added into the alignment algorithm, leading to very high accuracy of aperture alignment. When apertures are missed, the resulting increase in the background appears to be small [Fig. 7 ▸(*a*)] and the hit rates remain comparable to the silicon chips.

**Figure 10:**
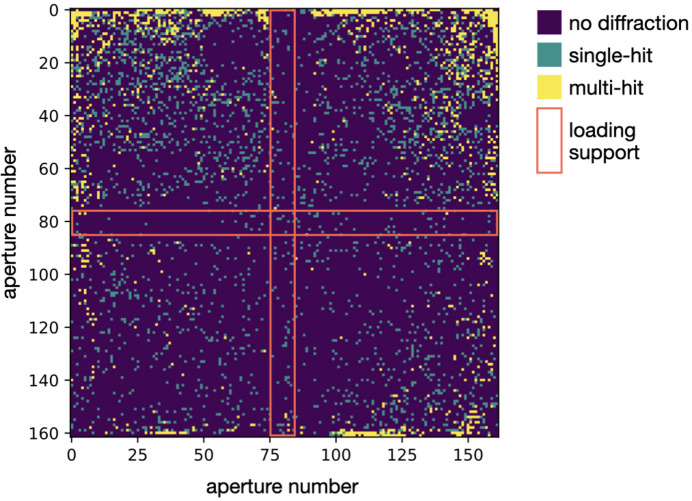
An example heat map of single- and multi-hit locations on a MISP chip. This map was generated using the 10 µm HEWL crystals loaded at a concentration of 5 × 10^5^ crystals ml^−1^. The red box indicates the cavities that were partially obscured by the supports in the vacuum loader.

During the hot embossing of the pyramid structures, the material of the film gets pushed to the outer edges of the pyramids, causing a substantial increase in film thickness. To compensate for this, pyramid heights were recalculated and modified on both master and working stamps to reach desired aperture sizes. Material drift also causes edge apertures to have an increased size as material is able to be distributed to the outer parts of the membrane. The larger edge apertures do not appear to affect the loading of crystals as high hit rates could be achieved when compared with the silicon supports [Figs. 4 ▸(*c*) and 4 ▸(*d*)]. It is possible that a higher density of multiple hits found towards the edges of the membranes is partly caused by these larger apertures (Fig. 10).

The intrinsic flexibility of the films used for membrane fabrication necessitated the attachment of a stabilization frame. This turned out to be an advantage as it allowed for larger volumes of solution to be loaded onto the surface of the chip, improving the crystal-loading efficiency (Fig. 4 ▸). The frame provided rigidity to the MISP chip, and allows the chip to be robust and user friendly. The principal disadvantage of the frame is that it necessitates an extra fabrication step and needs to be precisely fixed to the membrane. Wells can also be covered by the chip frame, which reduces the number of available chip apertures that can be exposed; this non-ideal solution will be refined in time in future chip iterations.

An essential piece of the loading process is the loading stage and setup. Initial trials of excess-mother-liquor extraction consisted of blotting paper where the main aim was to blot out the liquid from the back of the MISP chip. However, this was not efficient, resulting in excess mother liquor, high background and low hit rates. This led to the development of the loading stage, which was extremely beneficial as it provided an efficient method for extraction of excess mother liquor with the use of a vacuum pump. This improved the distribution of crystals, hit rate, background and provided an additional membrane support, protecting and prolonging the life of the membrane. Several types of proteins and crystals have been loaded on the chips and successfully tested during beam times, such as soluble proteins and membrane proteins, in the form of cubic-, rod- and plate-shaped crystals.

The MISP chips are reusable due to the stability of the polymer film, the rigidity provided by the frames and the loading stage. On average, a chip can be used 5–10 times without impacting crystal deposition, misalignment or increasing background noise, depending on protein type, crystal size, crystal concentration, solution viscosity, additives and formation of protein aggregates. To reuse the chips, they are soaked or sonicated in water or any polar solvent, and air dried afterwards.

To make better usage of limited beam times at X-ray sources, an even higher density of cavities on a chip might be desirable. For instance, going from a 120 to a 100 µm pitch would increase the density of cavities by more than 40%. Here, the current fabrication method poses limitations given by the angle of the pyramid sidewalls, defined by the KOH etching process of silicon and the film thickness. Reducing the pitch of the structures leads to smaller pyramids, which may only punch through films of smaller thicknesses. However, polymer films of similar quality and precise thicknesses in the range of 30–40 µm are not available off the shelf, and the production of dedicated films is very expensive given the low volume needed even for the production of several hundred chips. Production of pyramid arrays of higher aspect ratios might be feasible using non-silicon based microfabrication methods. However, it will be difficult to reach similar precision for the structures used as embossing stamp, in terms of positioning, sharpness of the tip and aspect ratios.

### 4.2. Opaque MISP chips for time-resolved serial crystallography

Pump–probe time-resolved serial crystallography (TR-SX) allows for observation of real-time changes in light-triggering systems (Moffat, 2001 ▸). In order to perform such experiments with the MISP chips, further developments were required. In fixed-target laser-pumped SFX, the key concern is ensuring that neighbouring wells are not contaminated by light. This is partly controlled by the size of the laser spot and the alignment of the laser and X-ray beam with respect to the chip. Efficient removal of excess mother liquor is also important. However, it is also critical to guarantee that light is not transmitted through the chip between wells. Due to this concern, a focus to explore opaque films was prioritized, resulting in the fabrication of black COC MISP chips.

Film production was carried out in-house since an opaque, thin and consistent COC or COP film is not commercially available. Methods such as film extrusion at a pilot plant were explored but, ultimately, solvent casting resulted in the most consistent thickness distribution for the film. The thickness range of films obtained in this process did play a slight role in the consistency of the aperture sizes in the membrane, causing a larger distribution of aperture sizes throughout the membrane. To further optimize the consistency of the aperture sizes of the black COC MISP chips, film extrusion or other methods of film production will be considered in the future.

The black film provides a barrier for the light used to illuminate specific wells not to trespass onto its neighbouring wells, inhibiting light contamination through the chip. It is now feasible to conduct laser-pump TR-SX experiments using the black MISP chips. Initial studies and results conducted with the black MISP chips will be presented in an upcoming article.

### 4.3. A question of crystal size

Our study into the effects of crystal size and concentration on fixed-target loading is essentially a more systematic continuation of those performed by Davy *et al.* (2019 ▸). The results show that single hits are far easier to attain for 25 µm crystals than for 5 µm crystals (Fig. 5 ▸). Based on first tests, this appears true, independent of crystal shape (*e.g.* rod- and plate-shaped crystals). Multi hits may not be a significant problem for static serial crystallography experiments such as radiation-damage-free structures, but they are not ideal in time-resolved pump–probe experiments.

In time-resolved experiments, protein molecules inside the crystal need to be triggered, typically by a pump laser but potentially also a substrate solution (Schulz *et al.*, 2022 ▸). The size and number of crystals in a fixed-target cavity will greatly affect the activation efficiency of a trigger as they will determine its penetration into the crystals. This determines the excited fraction of the proteins within the crystal and, hence, the observable signal in the diffraction pattern (Schmidt, 2013 ▸; Grünbein *et al.*, 2020 ▸). Small single crystals are ideal for such studies.

Our data on loading of the MISP chips, and in fact other micro-structured solid supports, suggest that the >30% hit rates promised by fixed targets are only possible for single crystals of the order of 25 rather than 5 µm. This is of particular concern for substrate-mixing experiments where <5 µm crystals are not only essential for the excited fraction but also for the activation time (Schmidt, 2013 ▸). It is possible that a different loading method, such as with an acoustic droplet ejector, can help resolve this problem (Davy *et al.*, 2019 ▸). However, it is also possible that an alternative chip design, specifically for use with small crystals, would help.

Modelling of serial crystallography hit rates is typically carried out using Poisson statistics (Park *et al.*, 2013 ▸; Hunter *et al.*, 2014 ▸). Anecdotally, these predictions never quite fit with the experience of actually using the apertured fixed targets, which tend to give a better ratio of single- to multi-hits than would have been predicted by a Poisson distribution. The explanation for this is that the crystals are no longer randomly distributed on a plane but funnelled into locations [Fig. 6 ▸(*b*)]. It is very possible that this feeling of non-conformity is primarily based on samples where crystals were >10 µm, and that users self-selected fixed targets for these types of samples.

The 5 µm crystals, by comparison with the 25 µm crystals, are much more randomly distributed upon the chip surface at 10^5^ crystals ml^−1^ or ∼400 crystals mm^−2^ [Figs. 6 ▸(*a*) and 6 ▸(*b*)]. At these concentrations, the 5 µm crystals do not appear to be drawn into the centre of the apertures. A future development here could be to increase the number of chip apertures by decreasing the thickness of the film, pitch size and cavity size, thereby increasing the number of focusing points for the crystals to funnel into as the mother liquor is removed by either blotting or vacuum.

The diffraction quality of the 5 µm crystals is also a slight concern given the reduction in observed resolution as measured by CC_1/2_ [Fig. 7 ▸(*b*)]. However, here an increase in the XFEL transmission is probably the simplest solution. The 50 µJ pulse used in all the commissioning experiments was initially chosen as the 25 µm HEWL crystals gave rise to overloads on the JUNGFRAU detector. This pulse energy represented a transmission of 10% of the beam. Given the lower resolution diffraction, an increase in transmission could probably be safely attempted.

### 4.4. Towards 100% hit rates

The promise of 100% hit rates has often been touted by many serial crystallography sample-delivery techniques. Fixed targets have, at least, presented a possible method to achieve this: crystal-location mapping. For the opaque silicon fixed targets, a method to achieve this has already been shown using absorption spectroscopy, albeit for a coloured protein (Oghbaey *et al.*, 2016 ▸). Therefore, the mapping of the silicon chips has currently failed to progress further.

Transparent polymer targets present the possibility of using light microscopy to find crystals located randomly on a mesh or encased between thin films (Martiel *et al.*, 2021 ▸). Problems can arise if crystals are able to move, but if imaging and software can be developed to reliably find crystals, mapping is possible. This is now also true for the transparent MISP chips.

Although microscopic inspection of areas did not match well with observed hits (Fig. 6 ▸), a more systematic automated approach could be successful. The main problem to overcome is the heterogeneous crystal loading (see Appendix *A*3[App appa]). If this problem is solved, the possibility for an aperture-aligned data collection with crystals in known locations can be realized. Then, empty apertures can be ignored, thus further increasing the project turnover of the endstation. For data collections using the SwissMX at Cristallina, this could considerably increase throughput. With the implementation of a robotic sample changer, six full-chip (23 × 23 mm chip) data collections per hour will be easily possible. If only wells with crystals were exposed, 9–10 chips per hour might be possible.

## 5. Conclusions

MISP chips were developed to enable aperture-aligned-style data collections in polymer-based fixed targets. From the results presented here, hot embossing of thermoplastics was shown to be a valid method to create cavities with well defined apertures at their bottom in a 50 µm thick polymer film. The resulting fixed targets were robust and preferable to other fixed targets and delivery methods, in terms of quantity and quality of data that can be recorded. There are still areas of these chips that require on-going optimization, particularly in sample loading and for 5 and 10 µm crystals. The 23 × 23 mm chip, however, has proven to be a good first iteration, and after expansion to 25 × 25 mm it will be the workhorse of the SwissMX endstation at Cristallina, SwissFEL, for experiments such as time-resolved pump–probe and substrate mixing over the next years. The chips are normally reusable for up to 5–10 times and could have a price point below that of similar silicon chips. However, the production is still complex and expensive due to the multi-stage silicon micro-fabrication and hot-embossing process. It may be possible to move towards a combination of roll-embossing and injection-moulding-style manufacturing processing, which after setup costs could drastically reduce the fabrication costs. Ultimately, it is hoped that the MISP chip can reduce the barriers to serial crystallography and further its reach in the structural biologist’s toolbox. 

## Supplementary Material

PDB reference: 5 µm HEWL crystals at room temperature, 8pyo

PDB reference: 10 µm HEWL crystals at room temperature, 8pyq

PDB reference: 25 µm HEWL crystals at room temperature, 8pyp

## Figures and Tables

**Figure 8:**
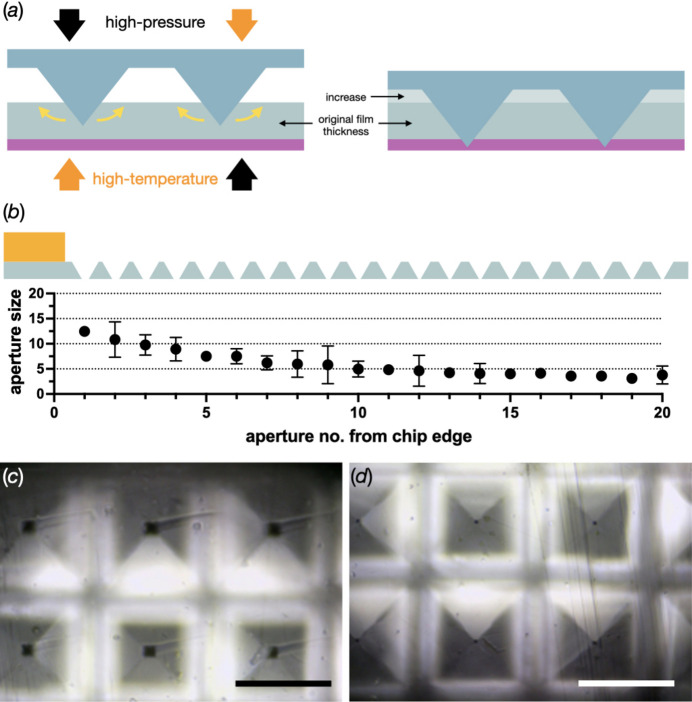
The effect of the material displacement from imprinting the pyramidal structures into COP film. (*a*) A scheme for depicting the process of how the films thicken as a result of the hot embossing. (*b*) A diagram and plot showing the gradual decrease of the aperture size as a function of the distance from the edge of the membrane. The error bars in the plot show the 95% confidence intervals. (*c*) and (*d*) Images of rows 1 and 2, and 19 and 20, from (*b*). The scale bar in both images is 100 µm.

**Figure 11:**
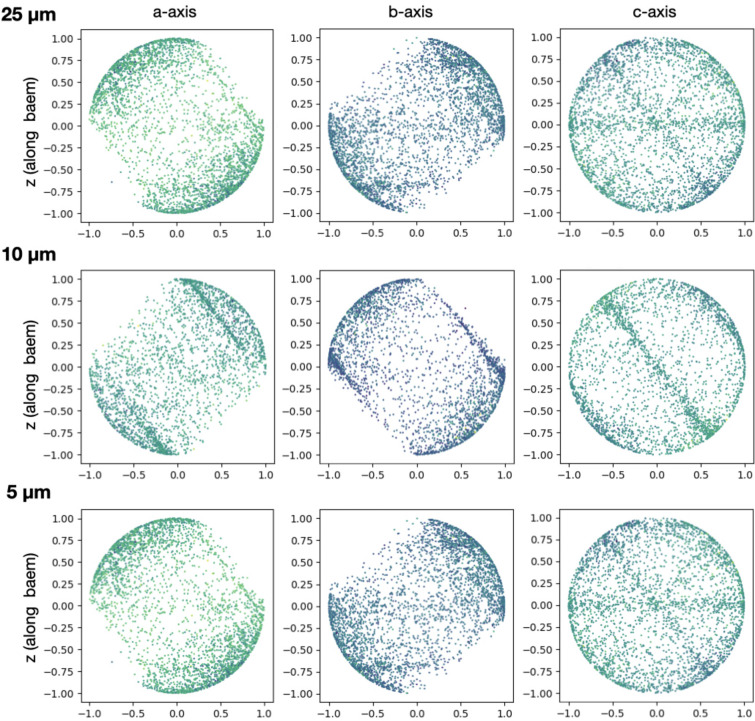
An analysis of the crystal orientation when loaded onto the MISP chips. Analysis on 25, 10 and 5 µm HEWL crystals was performed to observe the orientation of the crystals to the beam. Each point on the diagrams represents the crystal orientation on the *a*, *b* or *c* axis with respect to the beam.

**Figure 12:**
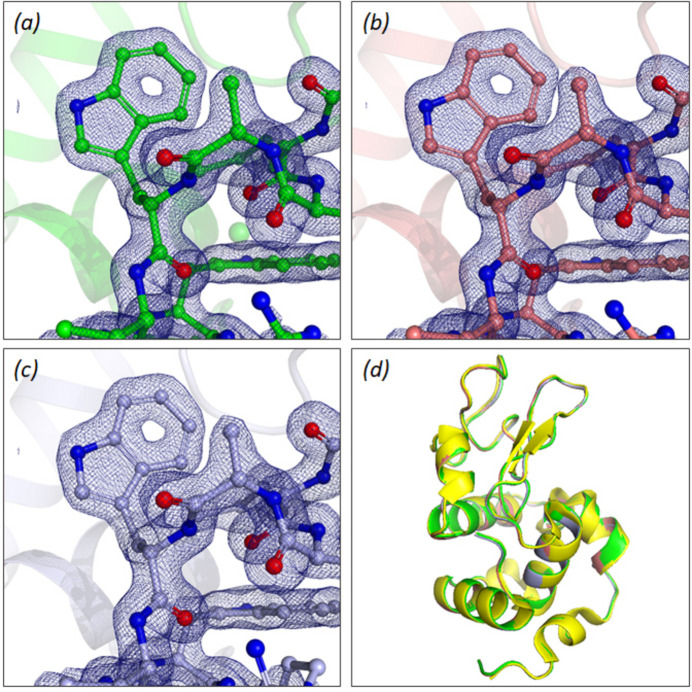
HEWL structures solved from different crystal sizes and alignment with a previously published HEWL structure. A section of the solved HEWL structure and electron density for (*a*) 25 µm crystals, (*b*) 10 µm crystals and (*c*) 5 µm crystals. Colours are consistent with previous figures. Electron-density maps are shown in dark blue to 1.5 Å. (*d*) Superposition of our HEWL structures for 25, 10 and 5 µm crystals with the previously solved HEWL structure, PDB ID 4n5r (Barends *et al.*, 2014 ▸) (yellow), displaying high uniformity between our structures and that previously collected under room-temperature conditions at an XFEL.

**Table 1:** Crystal sizes and concentrations grown from different HEWL solutions and crystallization-buffer concentrations All values are given as final concentrations after 1:1 mixing of protein and crystallization-buffer solutions.

HEWL (mg ml^−1^)	Crystallization buffer [%(*v*/*v*)]	Crystal (crystals ml^−1^)	Longest dimension (µm)
25.0	40	3.2 × 10^8^	5 ± 1
25.0	35	1.0 × 10^8^	10 ± 2
12.5	40	6.9 × 10^6^	25 ± 7

**Table 2:** Effect of imprinting pyramid arrays into 50 µm thick polymer films The periodicity (pitch) of the structures is 120 µm. Calculations were carried out for pyramids with bases ranging from 90 to 110 µm. The volume of the pyramids is a good estimate of the amount of polymer displaced leading to thickness increase. The punching height (fraction of the pyramid punching though the film) is equal to the thickness after thickness increase minus the pyramid height. The theoretical aperture size is given as the base of a pyramid of the same height.

Pyramid width (µm)	Pyramid height (µm)	Pyramid volume (µm^3^)	Film-thickness increase (µm)	Punching height (µm)	Aperture size (µm)
90	63.7	172054	11.9	1.8	2.5
95	67.3	202352	14.1	3.2	4.6
100	70.8	236013	16.4	4.4	6.3
105	74.3	273215	19.0	5.4	7.7
110	77.9	314134	21.8	6.1	8.7

**Table 3:** Data-collection and refinement statistics for the three HEWL structures

Dataset	5 µm	10 µm	25 µm
			
Data collection			
Space group	*P*4_3_2_1_2	*P*4_3_2_1_2	*P*4_3_2_1_2
Unit-cell parameters			
*a*, *b*, *c* (Å)	79.2, 79.2, 38.2	79.2, 79.2, 38.2	79.2, 79.2, 38.2
α, β, γ (°)	90, 90, 90	90, 90, 90	90, 90, 90
Indexed patterns	31659	29436	69420
Indexing rate (%)	5.1	5.01	66.98
Resolution range (Å)	25.05–1.54	39.60–1.40	25.05–1.40
Completeness (%)	100 (100)	100 (100)	100 (100)
Multiplicity	354 (229)	375 (231)	405 (218)
*R*_split_	0.021 (0.700)	0.025 (0.777)	0.027 (1.2)
CC_1/2_	0.943 (0.319)	0.977 (0.274)	0.982 (0.284)
〈*I*/σ(*I*)〉	5.67 (0.390)	6.25 (0.550)	7.34 (0.290)
*R*_work_	17.11	14.41	15.3
*R*_free_	20.15	17.86	19.66
Protein atoms	1100	1101	1084
Solvent atoms	112	91	107
RMS (bonds)	0.007	0.005	0.005
RMS (angles)	0.885	0.8	0.828
Ramachandran favoured (%)	99.21	99.21	98.43
Ramachandran outliers (%)	0	0	0
Mean *B* factor	26.01	28.43	28.56
Clashscore	2.77	1.84	2.34
PDB code	8pyo	8pyq	8pyp

## Appendix A

### A1. Film-thickness increase during hot embossing

During the embossing of pyramid structures, the material is squeezed towards the sides of the imprinting structures, increasing the average thickness of the polymer film (Fig. 8 ▸).

For the typically used parameter of 120 µm aperture pitch, and pyramids of 100 µm cavity size (71 µm height) imprinted into a 50 µm thick film, a theoretical thickness increase of ∼16 µm is calculated. This is well in line with the measured thickness increase of 14 ± 3 µm and apertures sizes of ∼5–7 µm, indicating that only the top 4–5 µm of the pyramids are penetrating the film.

Interestingly, increasing the pyramid dimensions at a constant pitch has only a minor influence on the aperture size. The increase in height is mostly compensated by an additional increase in film thickness (see Table 2 ▸). This in turn also indicates that the fabrication process critically depends on the change of film thickness during the hot embossing. This can be clearly observed when using a different thickness of polymer film. The change cannot simply be compensated by adapting the pyramid size linearly; all design parameters including the structure pitch have to be re-optimized.

The effect of film-thickness increase is more pronounced in areas of high structure densities such as the centre of the membranes and it levels out towards the borders of the structures. Fig. 8 ▸(*b*) shows the aperture sizes measured as a function of row number, starting at the edge of the membranes. Within the first rows, the aperture sizes steadily decrease starting at ∼12 µm, and level out at ∼4 µm in the 15th row [Figs. 8 ▸(*c*) and 8 ▸(*d*)].

In the areas of the fiducials, less material is displaced, leading to measured aperture sizes of ∼18 ± 2 µm for the 100 µm sized pyramids and the 50 µm COP film. In future designs, smaller pyramids will be used for the fiducials in order to increase the accuracy of positioning using the fiducials.

### A2. MISP-chip accessories

A loading station was constructed and found to be essential for sample loading onto the MISP chip. It consists of a loading platform, a vacuum pump to extract the excess mother liquor and a humidity stream to keep the crystals from dehydrating during preparation. Vacuum-pump suction was controlled with a valve, which provided control over the force and timing of excess-mother-liquor extraction (Fig. 9 ▸).

The loading platform was designed on *Fusion 360* and 3D printed on a ProJet MJP 2500 Series 3D printer [Fig. 9 ▸ (*b*)]. This loading platform provides a support for the membrane of the MISP chips whilst under suction, prolonging the lifespan of the MISP chips. The loading surface contains a PDMS gasket, providing assistance with creating a vacuum seal.

Prepared chips are transferred over to the sample holder designed on *Fusion 360* and 3D printed on a ProJet MJP 2500 Series 3D printer [Fig. 9 ▸(*c*)]. It contains two Mylar films to retain humidity inside the chip and is closed with two screws.

### A3. Crystal location on the chip after loading

Distribution of crystals on a MISP chip is partly understood by looking at an example bit-map of the hit locations on a chip (Fig. 10 ▸). The vacuum applied to the underside of the chip during loading appears to preferentially draw the solution and crystals towards the edges of the chip. This creates dense areas of multi-hits rather than a more homogeneous spread of single hits. The size of the loading-support footprint is also shown to be very large, obscuring some 3000 of the chip’s 29 584 available cavities. New versions will contain finer supports to reduce this issue.

### A4. Crystal orientations on the chips

Crystal orientation of HEWL crystals for all the measured datasets was extracted and evaluated using the *CrystFEL* stream files. Plots of the orientations are presented in Fig. 11 ▸. Data points are not randomly distributed into a homogeneous circle, as would be expected for random orientations. The gaps and asymmetric localization observed in the figure are indications of multiple crystals containing a similar preferential orientation. This small magnitude of preferential orientation is acceptable in the present case.

### A5. Structure determination

Structures were solved using the collected datasets of the three different crystal sizes (Fig. 12 ▸). Final refinement statistics are summarized in Table 3 ▸. All determined structures are in good agreement with literature data.
